# SGLT2 inhibitors and lower limb complications: the diuretic-induced hypovolemia hypothesis

**DOI:** 10.1186/s12933-021-01301-x

**Published:** 2021-05-13

**Authors:** Louis Potier, Kamel Mohammedi, Gilberto Velho, Ronan Roussel

**Affiliations:** 1 Centre de Recherche des Cordeliers, INSERM, Université de Paris, Sorbonne Université, Paris, France; 2 Hôpital Bichat, Assistance Publique Hôpitaux de Paris, Paris, France; 3 Department of Endocrinology, Diabetes and Nutrition, Bordeaux University Hospital, Hôpital Haut-Lévêque, Pessac, France; 4 Faculty of Medicine, University of Bordeaux, Bordeaux, France; 5 INSERM unit 1034, Biology of Cardiovascular Diseases, Pessac, France; 6 Service d’Endocrinologie Diabétologie Nutrition, Groupe Hospitalier Bichat - Claude Bernard, 46 rue Henri Huchard, 75877 Paris Cedex 18, France

**Keywords:** SGLT2 inhibitors, lower limb events, amputations, diuretics, hypovolemia

## Abstract

In a recent meta-analysis of randomized controlled trials of sodium glucose co-transporter 2 inhibitors (SGLT2i) in patients with diabetes, Lin and colleagues showed a positive association between SGLT2i-induced blood pressure and weight reduction and the risk of lower limb events. These results support the potential mechanism of a volume depletion effect of SGLT2i to explain the increase risk of amputation observed with this pharmacological class. Since the first result of the CANVAS trial raised a concern regarding the risk of amputation with SGLT2i, this hypothesis emerged from studies showing a higher incidence of amputations in patients with diabetes using diuretics. Furthermore, recent data found that copeptin, a surrogate marker of hydration status was also associated with lower limb outcomes. In conclusion, this assumption of diuretic-induced hypovolemia explanation highlights the fact that medications that induce a contraction of plasma volume, both traditional and novel agents with a diuretic mode of action should be introduced cautiously in patients with diabetes at high risk of diabetic foot events.

We read with great interest the Original Investigation by Lin and colleagues who performed an updated meta-analysis of randomized controlled trials of sodium glucose co-transporter 2 inhibitors (SGLT2i) in patients with diabetes [1]. The authors assessed associations between SGLT2i use and risk of lower-limb outcomes including lower-limb amputation (LLA), peripheral arterial disease (PAD) and diabetic foot events (DFE).

The results of the CANVAS trial showing excess risk of LLA in the canagliflozin arm versus placebo, raised significant concerns on the use of this new class of glucose lowering drugs in clinical practice [2]. This is particularly true in subjects suffering from previous or ongoing diabetic foot ulcers and/or PAD who show the highest risk of LLA. A large body of data was published on the subject, including observational, meta-analysis, pharmacovigilance and randomized studies [3]. All these studies have failed so far to elucidate the problem in a definitive way. However, in line with the results of Lin et al., the risk of LLA does not appear to be a class effect but rather related specifically to canagliflozin.

One of the most interesting and original aspect of this meta-analysis is the data regarding the association between blood pressure and weight reduction and the risk of lower-limb outcomes. The authors showed that greater weight and blood pressure (both systolic and diastolic) reduction induced by SGLT2i, were associated with higher risk of LLA, PAD and DFE. As extensively discussed by the authors, these results suggest that SGLT2i, through their diuretic effect, could induce volume depletion along with decreased tissue perfusion which then led to tissue necrosis and LLA. To support this diuresis-driven hypothesis, they mentioned the study by Erkens et al. showing that the use of thiazide diuretics increased the risk of LLA in population of subjects with type 2 diabetes [4]. We would like to bring to the attention of the authors and the readers some other data supporting this potential explanatory mechanism. First, two randomized controlled trials, the large Antihypertensive and Lipid Lowering Treatment to Prevent Heart Attack (ALLHAT) and the Intervention as a Goal in Hypertension Treatment (INSIGHT) trials showed a trend towards a higher rate of PAD-related events with chlorthalidone and co-amilozide respectively compared to non-diuretic blood pressure lowering treatments [5, 6]. In 2015, Gary et al. reported in cross-sectional observational study of 1000 participants with known PAD, a 1.5-fold increased risk of critical limb ischemia with use of loop diuretics [7]. More recently, we assessed this volume depletion hypothesis in a study comparing the risk of LLA according to the use of diuretics in patients with diabetes. Among 1459 patients with type 2 diabetes, we found a significant increased risk of lower-limb events (including LLA and lower-limb revascularization) in participants who were on diuretics (adjusted hazard ratio 1.49 [95 %CI, 1.01, 2.19; p = 0.04]) [8]. Thereafter, in the light of our previous study, we assessed the relationship between copeptin, a surrogate of vasopressin and therefore hydration status, and LLA in people with diabetes. We reported in cohorts of type 1 and type 2 diabetes that baseline plasma copeptin was significantly associated with LLA (adjusted HR 1.89 (1.28–2.82), p = 0.002 in type 1 diabetes and 1.42 (1.15–1.74), p = 0.001 in type 2 diabetes) [9]. Furthermore, our data were supported by a publication of Alshnbari et al. showing a 2.3-fold higher risk of LLA in diuretic-treated patients in a retrospective cohort study of 10,320 type 2 diabetes patients within the UK population (adjusted HR: 2.45; 95 %CI: 1.63–3.69). This association remained significant in the propensity score matched cohort [10].

To conclude, the study by Lin et al. added some new and interesting data to support the hypothesis raised by us and others that the risk of LLA associated with the use SGLT2i could be mediated by the diuretic-induced hypovolemia, which would worsen hypoperfusion of distal lower-extremities, triggering ischemia and necrosis, eventually leading to amputation. Even if recent studies from registries or randomized controlled trials provided reassuring data on the safety of SGLT2i, physicians should be aware of this potential mechanism and pay close attention to the diabetic foot events risk especially at the initiation phase where the volume depletion effect is more evident.

## Data Availability

Not applicable.

## Abbreviations

SGLT2i: Sodium glucose co-transporter 2 inhibitors
LLA: Lower-limb amputation
PAD: Peripheral arterial disease
DFE: Diabetic foot events.
CANVAS: CANagliflozin CardioVascular Assessment Study.
